# Psychiatrists should investigate their patients less

**DOI:** 10.1192/bjb.2021.125

**Published:** 2022-06

**Authors:** Matthew Butler, Fraser Scott, Biba Stanton, Jonathan Rogers

**Affiliations:** 1 King's College London, UK; 2 South London and Maudsley NHS Foundation Trust, London, UK; 3 King's College Hospital, London, UK; 4 University College London, UK

**Keywords:** Service users, in-patient treatment, imaging, ethics, cost-effectiveness

## Abstract

Psychiatrists often order investigations such as blood tests, neuroimaging and electroencephalograms for their patients. Rationales include ruling out ‘organic’ causes of psychiatric presentations, providing baseline parameters before starting psychotropic medications, and screening for general cardiometabolic health. Hospital protocols often recommend an extensive panel of blood tests on admission to a psychiatric ward. In this Against the Stream article, we argue that many of these investigations are at best useless and at worst harmful: the yield of positive findings that change clinical management is extremely low; special investigations are a poor substitute for a targeted history and examination; and incidental findings may cause anxiety and further unwarranted investigation. Cognitive and cultural reasons why over-investigation continues are discussed. We conclude by encouraging a more targeted approach guided by a thorough bedside clinical assessment.

The Royal College of Psychiatrists’ *Standards for Inpatient Mental Health Services* require a ‘comprehensive physical review’ to be initiated within four hours of admission to a psychiatric inpatient unit, which is to be completed within one week.^1^ In practice, this means that inpatients are often subjected to a battery of admission blood tests that are directed without regard for the presentation. Routine blood tests on admission remain a requirement in local trust policies,^2^ and clinical evaluations indicate that the majority of patients receive them.^3^ In addition, selected psychiatric patients may have further investigations, such as a magnetic resonance imaging (MRI) of the brain for those with a first psychotic episode, or an electroencephalogram (EEG) to rule out seizures.

In this article, we consider some of the reasons why investigations might be requested by psychiatrists and why they are often inappropriate, and suggest an alternative approach.

## The case for investigating

There are several (non-exhaustive) reasons why investigations might be requested for psychiatric patients:

- to screen for contributing physical factors in the acute presentation, including concern that rare but serious causes are not missed
- to provide baseline levels that may be relevant when starting particular psychotropic medications
- to screen for a range of chronic physical illnesses that might otherwise be missed because psychiatric patients may not present as often to healthcare professionals or are treated differently when they do
- to screen for health problems that are more common in those with severe mental illness, such as diabetes and liver disease.

Here, a distinction should be made between screening tests and diagnostic tests. Screening tests are those performed at population or subpopulation level to detect presymptomatic disease with the aim of early intervention. An example of this may be routine prediabetes screening in a patient on chronic antipsychotic treatment. Screening tests have been used in medicine since the middle of the 20th century (an early example being the successful screening and treatment of syphilis) and have undoubtedly improved diagnosis rates for medical and psychiatric patients.^4^ Despite this, there are a number of strict criteria that a screening test must meet before it is acceptable for use, including prior implementation of cost-effective primary prevention, a validated test, agreement on further investigation, good evidence for intervention at the presymptomatic phase, and evidence that benefits outweigh harms.^5^

Screening tests are distinct from diagnostic tests, which aim to accurately diagnose a condition for which there is clinical suspicion. An example of this may be positive antineuronal antibodies in the cerebrospinal fluid of a patient with clinically suspected autoimmune psychosis.

Although there are undoubtedly many cases where screening and diagnostic tests are entirely justified, we believe that investigations are often inappropriate because they have a low yield, poor sensitivity and specificity, limited interpretability without clinical correlation, and the potential to cause serious harm.

## The case for not investigating

### Tests may miss what they are looking for or not alter management

For an investigation to be useful, it should have high sensitivity (the ability to correctly identify patients with a given disease) and specificity (the ability to correctly identify patients without said disease). Distinct but related clinically useful concepts are those of positive predictive value (the probability that someone with a positive test has the disease) and negative predictive value (the probability that someone with a negative test does not have the disease). It is of note that even when tests do have high reported sensitivity and specificity, there may be additional complexities in real-world settings. This is in part because when developing tests, figures of diagnostic accuracy are calculated in a specific context, which is often obviously symptomatic individuals compared with obviously healthy controls.

Many diagnostic investigations used by psychiatrists fail to meet these criteria, and many have such a low probability of a clinically relevant result that it is hard to justify their routine clinical use. Systematic reviews have found that routine blood tests rarely alter the management of patients due for admission to psychiatric hospital, even when abnormal results are found.^6,7^ In one of the included studies of 500 patients, only one individual had an abnormal result that mandated urgent medical intervention. Notably, this patient was obviously symptomatic.^8^

Imaging and physiological investigations may not fare better. EEGs show interictal epileptiform discharges (IEDs) in only 29–55% of people with epilepsy,^9^ so it is inappropriate as a rule-out test for complex partial seizures presenting with psychotic symptoms. In contrast, EEG abnormalities (broader than just IEDs) occur in 19% of those treated with antipsychotics, so an abnormal EEG is not specific to a neurological cause of psychiatric symptoms.^10^ Similarly, one study found that 6% of healthy controls have at least one abnormality on an MRI of the brain, so the presence of an abnormality *per se* has a poor specificity for determining a neurological cause for psychiatric symptoms. This same research also concluded that none of 349 individuals with first-episode psychosis had MRI findings that necessitated a change in clinical management.^11^

### Investigations are no substitute for history taking and physical examination

Psychiatric patients invariably present in complex and myriad ways. In a minority, there is a recognisable physical cause for the psychiatric symptoms. For a physical illness to lead to psychiatric symptoms, there must either be a pathophysiological process (e.g. neoplastic, endocrine, autoimmune, metabolic, epileptiform, infective, neurodegenerative or metabolic) that is affecting the central nervous system and/or psychosocial mechanisms resulting from the physical illness. In each of these cases, it is very unlikely that a patient would present as entirely physically asymptomatic to an appropriately curious and observant clinician.

To put it another way, we might be surprised to see such physical illnesses presenting ‘occultly’, in the sense that a case would be missed after a thorough history and examination but picked up on a routine blood test or other untargeted investigation. Where psychosocial mechanisms predominate, these generally occur via experience of symptoms or via knowledge of a diagnosis, both of which can be elicited through an appropriate history or examination. In the specific case of functional disorders (such as functional neurological disorders), these are diagnosed through eliciting positive clinical signs, rather than being ‘diagnoses of exclusion’ which require investigation to exclude other causes.^12^

Even for tests with extraordinarily high sensitivity and specificity, there are further interpretative difficulties that arise in the absence of clinical suspicion. If a condition is very rare, then an abnormal investigation result may not be as diagnostic as is intuitively suspected. This is known as the ‘false-positive paradox’, or the ‘base rate paradox’, and occurs when the prevalence of a condition is lower than the test's false-positive rate (in these cases, the test will give more false positives than true positives). Crucially, the calculation is entirely different in populations who are already suspected to have the disease based on history and examination. In these cases, the pre-test probability (and thus the post-test probability after a positive test) would be much higher, and a resulting abnormal test much more clinically useful.

Take hypothetical blood test A which has a specificity of 99% for condition X. If condition X has a population prevalence of 1% and there are no additional features to increase the clinical suspicion of the condition (e.g. the patient has not been examined), we can assume the pre-test probability is 1%. In this case, an abnormal result from blood test A translates to a chance of having the condition (i.e. post-test probability) of only 50%. Most physicians, including us authors, would probably overestimate the post-test probability following a positive result on blood test A.

### Investigations can be harmful

Investigations often have attendant risks, which are seldom adequately considered. In some circumstances, taking blood can be painful or uncomfortable (particularly in a distressed, paranoid or disoriented patient), risking injury for patient and clinician. The noise and enclosed environment of an MRI scanner can be frightening for an anxious patient. Over-investigation may also contribute to the development or perpetuation of health anxiety, as it can serve to confirm a patient's fears about a serious undiagnosed illness.^13^ This may be particularly pertinent for individuals with somatoform or hypochondriacal disorders, who psychiatrists may encounter more often than might clinicians in other specialties.

A further important consideration is the impact of false positives. When tests are ordered in the absence of a clinical suspicion or hypothesis, a positive result inevitably returns the question: what next? In many cases, this might be further investigation, leading to a non-negligible potential of harm. Take the case of creatine kinase (CK), which is sometimes recommended as an admission blood test for psychiatric in-patients,^2^ despite being incidentally and benignly raised in many patients, and even being a poor discriminator for neuroleptic malignant syndrome.^14^ When faced with a raised CK in an asymptomatic patient, most of us feel that we should do something, so patients are frequently subjected to further blood tests and sometimes intravenous fluids for a test that should never have been done. More dramatically, if ten people are treated with endovascular coiling for an incidental unruptured intracranial aneurysm detected on MRI, one will end up disabled or dead within one year, all for a lesion that probably would not have caused any harm.^15^

Readers may be familiar with ‘incidentalomas’, an all-encompassing term for non-specific lesions seen on medical imaging. Incidentalomas have dubious clinical relevance, but often prompt further investigations and cause anxiety to patients and professionals alike. Relatedly, the phenomenon of non-specific abnormal results also occurs with blood tests. One study of healthy adults receiving a routine battery of blood tests found that over one-third had at least one abnormal result, of which only 7% were deemed to require a medical review (none of them urgently).^16^

Finally, in the context of limited healthcare funding, expensive investigations divert resources from more worthwhile causes. Discounting the cost of staff time, a simple routine battery of blood tests is likely to cost in the region of £15–20.^17^ The number of psychiatric admissions needed to screen to find a positive serum free thyroxine (FT4) is in the region of 127, which corresponds to a cost of around £400 per abnormal result.^18^ MRI brain scans cost in the region of £200. Cost-wise, there is likely more benefit from a five minute clinical history and examination.

## Cultural and psychological factors

Psychiatry is a medical discipline and there are, without any doubt, physical causes of psychiatric presentations. In addition, patients with severe mental illness are far more likely to die early, in part owing to huge over-representation of physical comorbidities such as cardiovascular disease. Psychiatrists should be attuned to physical health problems in in-patients as much as mental health problems, and we should feel confident in routinely performing thorough physical health histories and examinations.

The historical and ongoing divide between mental and physical healthcare is also likely to be a factor in over-investigation of psychiatric patients. Psychiatrists do not always have easy access to opinions from other specialties, and it is unusual for a physician to be integrated into psychiatric services. Psychiatrists may utilise unnecessary investigations when they are worried about patients, instead of being able to access a clinical opinion from specialist physicians. If physical and mental health services were better integrated, this could be avoided.

However, the overuse of investigations is a common theme in much of medicine and often functions more as a ‘psychological comfort blanket for clinicians’ rather than providing any tangible benefit to our patients.^19^ It may also stem from ‘addition bias’, the human tendency to try to solve challenging problems by adding something in rather than taking something away.^20^ Ordering an investigation may make us feel as though we have addressed the problem we were trying to solve, when in fact we might not have. For instance, if the problem is poor physical health outcomes in people with severe mental illness, admission blood tests are unlikely to be an answer. Focusing on this superficial attempt at joined-up healthcare perhaps distracts from other solutions, such as addressing stigma and reducing inequalities.

Furthermore, the familiar feeling that we must ‘do something rather than nothing’ may stem from a human tendency towards action, which is also known as intervention bias.^21^ Of course, as we have seen, there are scenarios where doing nothing is just as good, or even better, than doing something. The Hippocratic oath reminds us that our primary role as doctors is to first do no harm.

Overuse of investigations in psychiatry may also represent part of a wider risk-averse culture in medicine, which is sometimes referred to as ‘defensive medicine’. Defensive medicine is costly to healthcare institutions.^22^ Many doctors, including ourselves and three-quarters of all psychiatrists, admit to defensive medicine to some degree, including the ordering of clinically useless investigations.^23^ The reasons why clinicians may practise defensive medicine are myriad; however, the most commonly cited reason is fear of litigation resulting from malpractice hearings, particularly as the majority of cases of litigation stem from doctors missing diagnoses, rather than actively causing harm through the use of treatments.^22^ There are some (albeit incomplete) data that some aspects of defensive medicine may stem from physicians struggling to tolerate uncertainty in patient diagnosis and care.^24^

## Concluding remarks

We suggest six points to keep in mind when considering an investigation for our patients.

Limitations of tests:

1. Have we examined the patient and taken a history? Without a thorough examination, performing an investigation may not provide any useful information.
2. Is the disease common enough that this test will be useful? If the disease or condition is rare, there is a high probability of false positives.
3. How would we need to act if the test were to return an abnormal result? Additional interventions or investigations that may result could be harmful.

Limitations of human psychology:

1. Am I doing this test to resolve my own anxieties, or will it benefit the patient? Sometimes doing nothing rather than something is in the patient's best interests.
2. Can I tolerate the uncertainty of not knowing? Reaching after false certainty is not in anyone's interest.
3. Is there any chance the test or the results could lead to negative outcomes for the patient? First, do no harm.

We support attempts to integrate the body into mental health and illness. We believe that a good means of helping to achieve this is to ensure that we conduct thorough histories and examinations. This allows us both to request the appropriate investigations and to know how to interpret them once we have the results. This approach is supported by the American Psychiatric Association, which discourages routine laboratory testing,^25^ and by the National Institute for Health and Care Excellence (NICE), which does not recommend neuroimaging in first-episode psychosis.^26^ However, too often doctors are still faced with incidental findings on tests that should never have been requested. Psychiatrists should investigate their patients less and examine them more.

## Data Availability

Data availability is not applicable to this article as no new data were created or analysed in this study.

## About the authors

**Matthew Butler** is a Maudsley BRC Preparatory Clinical Research Training Fellow at the Institute of Psychiatry, Psychology & Neuroscience, King's College London, and a speciality trainee with South London and Maudsley NHS Foundation Trust, London, UK. **Fraser Scott** is a speciality trainee with South London and Maudsley NHS Foundation Trust, London, UK. **Biba Stanton** is a consultant neurologist at King's College Hospital, London, UK and South London and Maudsley NHS Foundation Trust, London, UK. **Jonathan Rogers** is a Wellcome Trust Clinical Training Fellow at University College London, UK.
